# Nutrient Intake Prior to Exercise Is Necessary for Increased Osteogenic Marker Response in Diabetic Postmenopausal Women

**DOI:** 10.3390/nu11071494

**Published:** 2019-06-30

**Authors:** Katarina T. Borer, Qingyun Zheng, Akram Jafari, Saba Javadi, Thomas Kernozek

**Affiliations:** 1 School of Kinesiology, The University of Michigan, Ann Arbor, MI 48109, USA; 2 School of Physical Education, Henan University Kaifeng, 475000 Henan, China; 3 Islamic Azad University Shahrekord Branch, 166 Shahrekord, Iran; 4 Department of Health Professions, Physical Therapy Program, University of Wisconsin –La Crosse, La Crosse, WI 54601, USA

**Keywords:** osteogenesis, markers of bone formation and resorption, nutrient intake, exercise and meal timing, HOMA-IR, parathyroid hormone, cortisol

## Abstract

Type 2 diabetes increases bone fracture risk in postmenopausal women. Usual treatment with anti-resorptive bisphosphonate drugs has some undesirable side effects, which justified our interest in the osteogenic potential of nutrition and exercise. Since meal eating reduces bone resorption, downhill locomotion increases mechanical stress, and brief osteogenic responsiveness to mechanical stress is followed by several hours of refractoriness, we designed a study where 40-min of mechanical stress was manipulated by treadmill walking uphill or downhill. Exercise preceded or followed two daily meals by one hour, and the meals and exercise bouts were 7 hours apart. Fifteen subjects each performed two of five trials: No exercise (SED), uphill exercise before (UBM) or after meals (UAM), and downhill exercise before (DBM) or after meals (DAM). Relative to SED trial, osteogenic response, defined as the ratio of osteogenic C-terminal propeptide of type I collagen (CICP) over bone-resorptive C-terminal telopeptide of type-I collagen (CTX) markers, increased in exercise-after-meal trials, but not in exercise-before-meal trials. CICP/CTX response rose significantly after the first exercise-after-meal bout in DAM, and after the second one in UAM, due to a greater CICP rise, and not a decline in CTX. Post-meal exercise, but not the pre-meal exercise, also significantly lowered serum insulin response and homeostatic model (HOMA-IR) assessment of insulin resistance.

## 1. Introduction

Type 2 diabetes (T2D) compounds the vulnerability of the skeleton to bone fracture in postmenopausal women. Postmenopausal estrogen decline leads to bone mineral loss, and lower bone mineral density (BMD) leads to osteopenia and osteoporosis [1] with an associated increase in bone fractures [2]. T2D produces a different effect in that it increases the risk of bone fractures [3] independently of bone mineral density [4,5] apparently due to a deficit in bone quality [5]. While the reasons for changes in diabetic bone quality are not fully understood, they could be a consequence of advanced glycation end products or other pathologies [6], but may also be the result of reduced nutrient access to the bone tissue due to systemic insulin resistance [7], the key characteristic of T2D [8]. Impaired insulin signaling in diabetes is postulated to cause a deficit in mineralized bone surface area, a decrement in the rate of mineral apposition, deceased osteoid surface, depressed osteoblast activity, and decreased numbers of osteoclasts [9]. Aging as a part of the transition into menopause is also associated with reduced capacity of bone formation and increased propensity to bone resorption [10,11]. 

Bone vulnerability to fracture due to menopause [12] or diabetes [13] has been mostly treated pharmacologically with anti-resorptive bisphosphonate drugs. Prolonged treatment with these drugs has been reported to occasionally increase the incidence of femoral fractures [14] and osteonecrosis of the jaw [15]. This prompted our interest in the use of an appropriate combination of exercise and nutrition as a substitute for, or adjunct to, pharmacological approaches to preventing osteoporotic fractures in postmenopausal diabetic women. To the best of our knowledge, the feasibility of using a combination of exercise and nutrition to improve diabetic bone health has not been systematically examined to date.

Exercise of supra-threshold strain of either high amplitude and low frequency such as locomotion, running, and jumping [16,17], or of low amplitude and high frequency such as vibration [18,19] has been shown to promote bone mineral accretion in bone segments resisting the strain [20]. Exercise is also generally known to increase glucose uptake in tissues by a mechanism independent of insulin action [21], so that it can improve tissue nutrient uptake even in the presence of systemic insulin resistance. Nutrient intake was reported to reduce blood concentration of a marker of bone resorption coincidentally with the increase in a gut peptide GLP2 (glucose-dependent insulinotropic peptide 2) in a meal-associated diurnal pattern, with higher resorption during sleep, and reduced resorption during daytime prandial periods [22]. Mediation by GLP2 of meal-associated reductions in bone resorption was suggested by reduced resorptive bone marker response after experimental administration of this peptide [22].

The aim of this study was to determine whether an adequate mechanical stimulus to the skeleton through loading, and the appropriate timing of meals, could improve the balance of bone formation to bone resorption in postmenopausal diabetic women. In designing the study, mechanical skeletal loading was enhanced by downhill treadmill walking and contrasted to unloading during uphill walking. The idea regarding loading was based on a study where running at 3 m/sec at a 6° decline increased ground reaction force (GRF) by 24.3%, while running on a +6° incline reduced it by 22% relative to exercise on level surface [23]. We therefore manipulated in this study the magnitude of GRFs by providing the treadmill grade at either −6° or +6°. We also manipulated the magnitude of cardiovascular effort by having women walking uphill exercise at 75% of $\dot{V}$O_2max_ and walking downhill at 46% of $\dot{V}$O_2max_. The 75% maximal effort was selected because postmenopausal women walking 4.8 km per day at that intensity on a level surface 4 days a week for 15 weeks increased the areal BMD of their legs and whole body, but not of other skeletal sites [24]. A threshold load of 872 N (1.22 times body weight) was necessary to prevent BMD loss and produce a gain, while walking at the lower intensity did not have this effect. The utilization of a lower cardiovascular effort at 46% $\dot{V}$O_2max_ was also selected in the present study to counterbalance the greater mechanical loading with downhill exercise and to make downhill walking more comfortable.

The insight regarding the brief efficacy of the bone anabolic response to a suprathreshold mechanical load [25,26] led us to provide 40 minutes of exercise. The insight that the bone is refractory to repeat mechanical stimulation over a period of 6 to 8 hours and demonstrates greater bone formation when the same amount of loading is spaced over such an interval rather than provided in one loading bout [25,27,28], prompted our separating the two daily meals and exercise bouts 7 hours apart. The information that meal taking lowers markers of bone resorption [22] has prompted our provision of exercise within 80 minutes before or after the meals to test the temporal sensitivity of the loaded bone to absorbed nutrients. We measured mechanical loading during exercise with mechanosensitive in-shoe sensors, and also monitored psychological ratings of perceived exertion (RPEs), and the heart rate as a physiological measure of stress, as such stress can affect hormonal environment and influence bone responses. To assist in making inferences on the possible hormonal interactions with mechanical, cardiovascular, and psychological stress in our study, and thus their contribution to changes in bone markers and insulin resistance, we added measurements of plasma concentrations of parathyroid hormone (PTH) and cortisol to the measurements of insulin and glucose. PTH measurements were prompted by the known role of this hormone’s pulsatile secretion during exercise or administration in promoting bone formation [28,29], the fact that PTH secretion during exercise was reported to increase in both men [30] and postmenopausal women [31], and the success of reducing the risk of osteoporosis with intermittent administration of the PTH analog Teriparatide [32]. Cortisol was measured because it is a marker of physiological and psychological stress [33] and because of its bone resorption promotion [34]. Insulin and glucose measurements were included to assess the possible role of reduced nutrient access to the bone as a cause of low diabetic bone quality, as the measurements allow assessment of insulin resistance in meal-tolerance tests [35].

We entertained three hypotheses: (1) Higher GRFs generated by downhill walking would produce greater osteogenic response, defined as the balance or ratio between a marker of bone formation and a marker of resorption when compared to lower GRFs generated by uphill walking; (2) the loading stimulus of exercise would produce an osteogenic effect that would operate for at least 80 pre-meal minutes through the immediate postprandial period; and (3) the osteogenic response will predominantly reflect a decline in the marker of bone resorption rather than an increase in the marker of bone formation because of the advanced age of postmenopausal diabetic women. 

## 2. Materials and Methods 

### 2.1. Subjects 

Fifteen postmenopausal women with T2D were recruited from the University of Michigan clinical studies web page (UMClinicalStudies.org). Inclusion criteria were 50 to 65 years old, surgical or natural menopause (no menstrual periods for at least one year), medical diagnosis of T2D but of no other metabolic disease, body mass index (BMI) of 25 to 35 kg/m^2^, no exposure to hormone replacement therapy (HRT), non-smoker, absence of musculo-skeletal disabilities that would impair walking, and sedentary status (<60 minutes of regular exercise per week). Diabetes treatment entailed daily intake of metformin (1000 mg in 11 subjects and between 1500 and 2000 mg in four others) and additional glycemia-lowering drugs in 3 subjects. Ten subjects, each, received cholesterol-lowering and hypertension drugs, and three were also treated for depression. All subjects signed an informed consent for human clinical studies before admission to the study. The study was conducted in accordance with the Declaration of Helsinki, and the protocols were approved by the University of Michigan Institutional Review Board (HUM 32227 on 10/8/2009, and HUM 32700 on 12/9/2009). These constituent protocols were registered as a clinical trial NCT03930758 with Clinical Trials.gov after the initiation of subject recruitment in 2009. All subject recruitment was completed by September of 2012. All trials for this study were registered.

### 2.2. General Experimental Protocol

Subjects underwent preliminary health and fitness screens at the Michigan Clinical Research Unit (MCRU). The health screen included health history, measurements of weight, height, and body fat by a dual-energy X-ray absorptiometry apparatus (model Prodigy, Lunar Radiation Corporation, Madison, WI, USA), and a fasting blood draw for fasting glucose and other laboratory chemistries. A preliminary fitness screen assessed individual maximal aerobic effort. It consisted of a treadmill test at 4.8 km per hour with 2% slope increments every 3 minutes. To obtain oxygen consumption ($\dot{V}$O_2_) and carbon dioxide production ($\dot{V}$CO_2_), the subject was breathing through a mouth piece into a gas meter using a Max II metabolic cart (AEI Technologies, Inc., Bastrop, TX, USA). Gas meter calibration was done with pre-calibrated gas tanks. The criterion for maximal effort used was a respiratory quotient (ratio of $\dot{V}$CO_2_/ $\dot{V}$O_2_) of 1. After matching by age, body weight, BMI, and aerobic fitness, subjects were randomly assigned to two out of five trials: A sedentary no-exercise trial (SED), two downhill trials at a −6° treadmill decline, one before the two daily meals (DBM) and the other after meals (DAM), and to two uphill trials at +6° treadmill incline, one before the meal (UBM) and the other after eating (UAM).

### 2.3. Study Design

A week after the fitness test, subjects were admitted to the MCRU at 06:30 for a 24-hour trial. At 06:45, an intravenous catheter was inserted into an antecubital vein. At 07:00, they received 1 g calcium and a 600 IU vitamin D supplement with one oz of orange juice. Two weight-maintenance meals were provided at 10:00 and 17:00. Exercise before the meals was performed from 08:00 to 08:40 and 15:00 to 15:40 (UBM and DBM trials) and from 11:00 to 11:40 and from 18:00 to 18:40 after the meals (UAM and DAM trials). During exercise, women wore dynamic in-shoe-pressure insoles containing sensors that provided information on peak pressures exerted during walking, and heart-rate-monitor chest bands and wrist watches (Polar Electro, Bethpage, NY). Blood (5 ml) was collected hourly between 08:00 and 20:00, at midnight, and at 06:00 the next morning.

### 2.4. GRF Manipulation and Measurements

**Figure 1:**
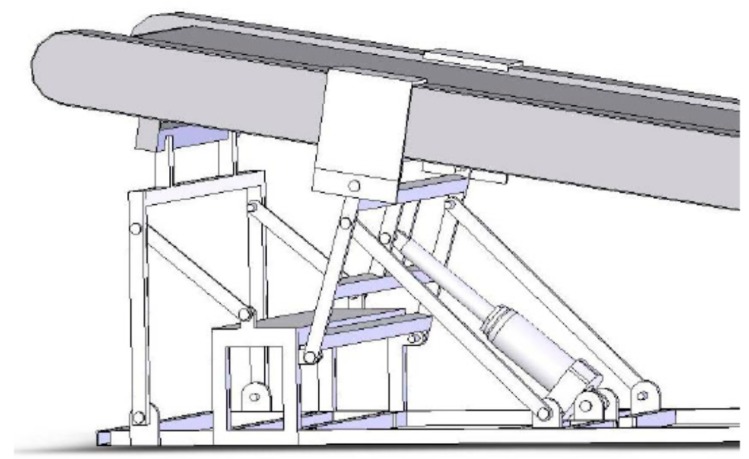
Treadmill elevator. A lever arm, powered by a mechanical jack, raises the rear end of the treadmill to a −6° slope.

Uphill treadmill slope elevation was used to reduce GRFs and downhill treadmill slope to increase GRFs relative to level walking [23]. Downhill slope adjustment was accomplished through a treadmill modification, which entailed construction of a lever arm powered by a mechanical jack that raised the rear end of the treadmill to create a −6° angle treadmill slope (Figure 1).

Ground-reaction force (GRF) measurements normal to the plantar surface during walking were recorded during each stepping cycle bilaterally at 50 Hz using the in-shoe pressure-measuring insoles (Novel Pedar, Novel Electronics, St Paul, MN, USA) and the associated computer software (Pedar Professional, Novel Electronics, St Paul, MN, USA). The mechanosensing apparatus, carried on a belt around the patient’s waist, was calibrated before each trial using the computer program provided by Novel Pedar. The pressure measurements were captured by BlueTooth technology to a Novel Pedar computer program. The last 6 minutes of the 40-minute exercise measurement was used to determine peak pressure (in kilo-pascals, KPa) and ground reaction force (GRF in Newtons, N) for each trial. GRFs were derived from a sum of pressures from all of the insole sensors.

### 2.5. Exercise Intensity

For the uphill walking exercise, a slope of +6° was used, and for downhill exercise, a slope of −6°, and the specific treadmill speed was adjusted to achieve respective target exercise intensities of 75% and 46% of maximal effort. Desired intensities were estimated during the first 10 minutes of exercise from the preliminary fitness tests and measured and adjusted using respirometry with the metabolic cart during the remaining 30 minutes of the bout.

Intensity-associated stress during exercise was measured with Borg’s rating of perceived exertion (RPE) scale [36]. The scale ranges from 6 to 20, and seven intensities are identified as: 7 = very, very light, 9 = light, 11 = fairly light, 13 = somewhat hard, 15 = hard, 17 = very hard, and 19 = very, very hard. RPEs, along with heart rates, were measured both during the preliminary aerobic fitness test and during exercise trials at 5-min intervals. 

### 2.6. Meals

Two weight-maintenance meals were provided at 10:00 and 17:00 h. Macronutrient composition was 60% carbohydrate, 15% protein, and 25% fat. Foods to meet this composition were selected by MCRU dieticians. The morning meal included egg salad plate with multi grain bun, wheat roll with margarine, coleslaw, carrot sticks, skim milk and orange juice, graham crackers, and a serving of fresh fruit. The evening meal included a sandwich composed of 2 slices of bacon, 1 slice of American cheese and 2 oz. of baked ham with green-leaf lettuce, wheat toast with diet mayonnaise, cooked broccoli, cauliflower, and carrots, tossed salad with diet French dressing, pretzels, 1.5 serving of fresh fruit, a carton of cranberry cocktail, and vanilla ice cream.

### 2.7. DXA Measurements

Bone mineral content and body composition scans were performed with dual energy X-ray absorptiometry (DXA) scanner (model Prodigy, Lunar Radiation Corporation, Madison, WI, USA) using the pencil beam mode. Bone regions scanned were lumbar spine (L2 through L6), femoral neck, trochanter, Ward’s triangle, and femoral shaft for determination of areal BMD (g/cm^2^). Coefficients of variation (CVs) for BMD measurements of the separate regions ranged between 1.5% (spine) and 2.0% (hip). The quality control program included weekly calibration studies.

### 2.8. Blood Collection

Blood samples were collected into serum-separation tubes containing spray-coated silica and polymer gel for serum separation (BD Vacutainer venous serum separation tubes: Hemogard, Fisher Scientific, Pittsburg, PA, USA) for determination of bone markers, glucose, insulin, PTH, and cortisol. After about 15 minutes in serum-separation tubes, serum was separated by centrifugation at 2000 g and stored at −80 °C for later hormone and bone marker determinations.

### 2.9. Markers of Bone Formation

CICP (C-terminal propeptide of type I collagen), a marker of bone formation, and CTX (C-terminal telopeptide of type-I collagen), a marker of bone resorption, were measured with enzymatic immunoassay using kits provided by Quidel (Santa Clara, CA, USA). Metra-CICP enzyme immunoassay had a sensitivity of 0.2 ng/ml. Intra- and inter-assay coefficients of variation (CVs) at three dose levels ranged between 5.5% and 7%. CTX was measured with serum CrossLaps enzyme immunological assay by Nordic Bioscience Diagnostics (also supplied by Quidel). Sensitivity of this assay was 20 pg/ml. Intra- and inter-assay CVs at three dose levels ranged between 5.0% and 8.1%.

### 2.10. Hormone Measurements

Intact PTH (DiaSorin, Vercelli, Italy) was measured in four subjects per group with solid-phase two-site chemiluminescent immunometric assay. Intra- and inter-assay CVs for PTH at two dose levels were between 1.2% and 2.2% and 4.8% and 7.7%, respectively. Cortisol was measured by a solid-phase radioimmunoassay (Siemens Medical Solutions Diagnostics, Los Angeles, CA). Intra- and inter-assay CVs for cortisol were between 3% and 5.1%, and 4% and 6.4%, respectively.

### 2.11. Statistical Analyses

Data are presented as means and SEMs. As bone markers, the osteogenic ratio, glucose, insulin, PTH, and cortisol exhibited changes during the two postprandial (PP) periods, areas under the curve (AUCs) were calculated by the trapezoidal rule for the morning PP (10:00 to 17:00) and the afternoon PP (17:00 to 0 h). To eliminate the significant initial inter-group differences in bone markers, PTH and cortisol, the results for these variables were expressed as percent change. A ratio between CICP, marker of bone formation, and CTX, marker of bone resorption, was used as a measure of the osteogenic response in comparing the effects of the four exercise trials to the sedentary trial. Subject characteristics, trial respiratory and GRF measurements, and initial serum concentrations of bone markers and hormoneswere evaluated with one-way ANOVA. Mixed-model ANOVA was used to analyze the effects of timing of meals and exercise with Statistical Analysis System software (SAS version 9.3, SAS Institute, Cary, NC, USA). Postprandial AUCs in the five trials, for morning and afternoon combined, or individually, were analyzed as between-subject effects, and the values for each of 15 subjects, as within-subject random intercept. Tukey–Kramer post-hoc analyses evaluated between group differences with adjustment for multiple comparisons. Insulin resistance during PP periods within each of five trials was estimated with the homeostatic model assessment (HOMA-IR) procedure [37] for the meal tolerance test [35] validated against the minimal model and the intravenous glucose tolerance test [38]. To calculate HOMA-IR, the product of insulin and glucose AUCs was divided by 405. Figure graphics were performed with GraphPad Prism 8.1 software (San Diego, CA, USA).

## 3. Results

Of the 15 subjects, 12 were Caucasian and 3 were African American. Non-Caucasian subjects were represented in all but the UBM trial. There were no group differences in any of the 17 variables compared before the start of the study (Table 1).

### 3.1. Subject Characteristics (Table 1)

**Table 1:** Subject characteristics.

Variable	Sedentary	Uphill Before Meals	Downhill Before Meals	Uphill After Meals	Downhill After Meals	*F*; *p*
Subjects	*N* = 6 (3 AA)	*N* = 6 (6 C)	*N* = 6 (1 AA)	*N* = 6 (1 AA)	*N* = 6 (1 AA)	
Age (years)	58.5 ± 1.8	56.7 ± 1.7	56.5 ± 1.4	56.5 ± 1.8	56.2 ± 1.8	0.294, 0.88
Menopause (y)	10.5 ± 2.8	5.2 ± 1.0	4.7 ± 1.3	6.0 ± 2.5	7.3 ±2.5	1.14,0.36
Diabetes (years)	11.0 ± 1.6	10.8 ± 1.4	11.3 ± 4.5	9.2 ± 2.7	7.4 ± 2.5	0.191, 0.94
Glucose (mg/dl)	122.5 ± 12.02	163.17 ±35.93	164.67 ± 35.25	118.50 ± i9.25	125.17	0.926, 0.46
PTH (ng/ml)	43.83 ± 3.79	38.50 ± 5.45	37.83 ± 4.92	40.83 ± 2.44	46.00 ± 3.52	0.701, 0.60
TSH (ng/ml)	2.06 ± 0.52	2.53 ± 0.80	2.01 ± 0.80	1.83 ± 0.46	2.10 ± 0.53	0.162, 0.96
Weight (kg)	70.57 ± 4.15	66.95 ± 4.46	71. 35 ± 4.52	73.20 ± 4.25	69.03 ± 4.37	0.296, 0.88
BMI (kg/m^2^)	27.26 ± 1.39	26.13 ± 1.45	26.43 ± 1.11	26.75 ± 1.08	26.65 ± 1.36	0.106, 0.98
Body fat (%)	39.97 ± 1.37	39.78 ± 2.24	37.75 ± 1.07	39.5 ± 1.01	41.00 ± 2.05	0.524, 0.72
BMD, body (g/cm^2^)	1.23 ± 0.07	1.16 ± 0.08	1.26 ± 0.05±	1.21 ± 0.06	1.19 ± 0.05	0.379, 0.82
Z score, body	1.63 ± 0.62	1.15 ± 0.48	2.08 ± 0.49	1.48 ± 0.61	1.42 ± 0.48	0.406, 0.80
Spine BMD (g/cm^2^)	1.18 ± 0.08	1.11 ± 0.07	1.25 ± 0.05	1.19 ± 0.07	1.12 ± 0.07	0.459, 0.76
Spine Z score	0.78 ± 0.51	0.25 ± 0.43	0.98 ± 0.37	0.80 ± 0.45	0.38 ± 0.47	0.476, 0.75
Hip BMD (g/cm^2^)	1.06 ± 0.08	0.99 ± 0.08	1.05 ± 0.08	1.02 ± 0.05	1.05 ± 0.07	0.163, 0.96
Hip Z score	0.88 ± 0.52	0.62 ± 0.59	0.95 ± 0.58	0.73 ± 0.41	1.28 ± 0.47	0.241, 0.91
VO_2 max_ (ml O_2_/min)	1301.72 ± 130.42	1371.70±139.16	1578.78 ± 210.98	1587.88 ± 227.93	1398.92 ± 131.32	0.552, 0.70
VO_2 max_ (ml/kg min)	18.48 ± 1.56	20.32 ± 1.15	21.75 ± 1.67	21.48 ± 2.33	20.17 ± 1.21	0.625, 0.65

BMI = body mass index, TSH = thyroid stimulating hormone, Z score = number of standard deviations below the bone minreal density mean score normalized for age.

### 3.2. Exercise Parameters (Table 2)

Both the application of different GRFs and exercise intensities were effective (Table 2). Relative cardiorespiratory intensity in the two uphill groups was higher at 75.2% (UBM trial) and 76.3% of maximal effort (UAM trial) (F_df 4,25_ = 50.43, *p* < 0.0001) than in the two downhill trials where the relative effort was between 48.1% (DBM trial) and 47.6% (DAM trial). On the other hand, both absolute and weight-normalized peak GRFs and relative peak pressures were higher during the two downhill trials (F_df 4,25_ = 8.7, *p* = 0.0007. F_df 4,25_ = 25.6, *p* < 0.0001, and F_df 4,25_ = 3.883, *p* = 0.0025, respectively) than during the two uphill trials. Peak GRFs, both absolute and normalized to body weight, were about 33% to 34% higher in downhill compared to uphill trials, and the relative peak pressure was 20% higher. RPE values of subjective stress were not different in the four exercise groups. The ratings ranged between light (10) and somewhat hard (12) for all exercise groups. Trial heart rates were variable (F_df 4,25_ = 5.60, *p* = 0.0057). Both the RPE and HR values appeared highest in the UAM trial, but there were no between-trials differences in either variable after Bonferroni correction. 

**Table 2:** Exercise parameter results.

Variable	Uphill Before Meals	Downhill Before Meals	Uphill After Meals	Downhill After Meals	*F* (df = 3,20); *p*
Subjects	*N* = 6 (6 C)	*N* = 6 (1 AA)	*N* = 6 (1 AA)	*N* = 6 (1 AA)	
Relative effort (%)	75.15 ± 0.89	48.13 ± 3.83	76.33 ± 2.0	47.60 ± 1.16	*F* = 50.43; *p* < 0.0001
RPE	11.11 ± 0.73	9.95 ± 0.71	11.95 ± 0.77	11.22 ± 0.92	*F* = 1.105, *p* = 0. 370
HR (bpm)	114.73 ± 4.36	103.65 ± 7.14	130.58 ± 2.69	113.72 ± 3.25	*F* = 5.60, *p* = 0.0057
Peak GRF (N)	857.50 ± 52.80	1104.72 ± 40.79	795.27 ± 36.50	1105.00 ± 79.71	*F* = 8.732, *p* < 0.0007
Relative GRF (N/kg)	1.31 ± 0.02	1.59 ± 0.05	1.12 ± 0.04	1.64 ± 0.07	*F* = 25.60, *p* < 0.0001
Peak pressure (KPa)	257.68 ± 22.80	284.96 ± 18.13	225.08 ± 25.48	301.73 ± 37.69	*F* = 1.541, *p* = 0.235
Relative pressure (KPa/kg)	0.39 ± 0.02	0.41 ± 0.02	0.32 ± 0.03	0.44 ±0.04	*F* = 3.883, *p* = 0.00245

HR = heart rate, GRF = ground reaction force.

### 3.3. Bone Marker Measurements

Starting absolute serum values of bone markers showed great residual-to-treatment variances. For CICP, the starting concentrations were 130.2 ± 31.1 for SED, 150.1 ± 20.7 for UBM, 101.2 ± 23.3 for DBM, 126.1 ± 24.7 for UAM, and 151.1 ± 21.5 ng/ml for DAM trial, and the residual variance was 14.2 times greater than treatment variance. Corresponding values for CTX were 0.65 ± 0.12 for SED, 0.69 ± 0.15 for UBM, 0.58 ± 0.14 for DBM, 0.72 ± 0.17 for UAM, and 0.77 ± 0.14 ng/ml for DAM trial, while the residual variance was 22.75 times greater than treatment variance. Therefore, serum bone markers for exercise versus sedentary trials were presented as percent changes.

#### 3.3.1. CICP

**Figure 2:**
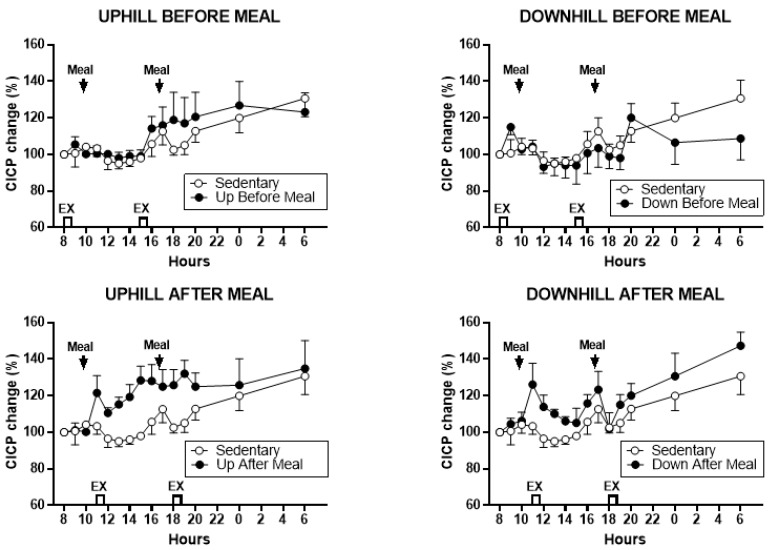
Percent changes in serum C-terminal propeptide of type I collagen (CICP) between sedentary trial and exercise trials performed before meals is shown at the top (left: uphill exercise before meal (UBM), right: downhill exercise before meal (DBM)), and after exercise trials performed after eating, is shown at the bottom (left: uphill exercise after meal (UAM), right: downhill exercise after meal (DAM)). CICP rose relative to no exercise (SED) trials in both exercise-after-meal trials (lower panels) and showed little change in the two exercise-before-meal trials (top panels).

**Figure 3:**
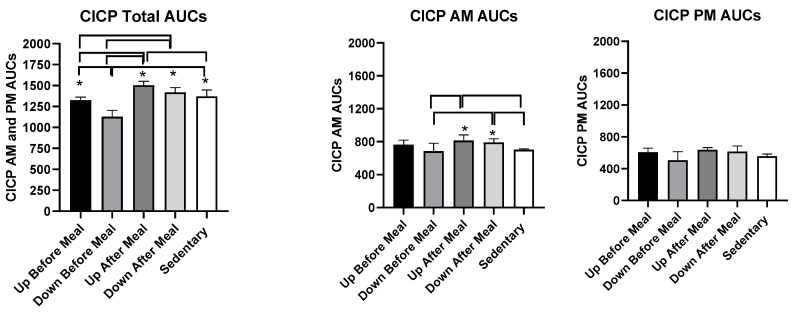
Postprandial CICP areas under the curve (AUCs) after the morning and afternoon meals with the two AUCs combined (left) and the morning (center) and afternoon (right) AUCs shown individually. Greatest increases in CICP AUCs were seen in the two exercise-after-meals trials (UAM and DAM) relative to sedentary trials, both for combined AUCs (left) and morning postprandial AUCs (center). Only UAM CICP AUC remained higher than the SED trial after the afternoon meal. Combined CICP AUC in the UBM trial also was significantly higher than in the DBM trial. * indicates significant difference relative to groups marked by the overhead bracket.

Treatment effects on CICP percent change are shown in Figure 2, and analysis of treatment contrasts between the CICP AUCs in exercising and SED trials in Figure 3 and Table 3.

With morning and afternoon AUCs combined, there were significant differences between CICP trials (*F*_(df 4,11)_ = 16.26, *p* = 0.0001). As shown in Figure 3, bottom left and Table 3, the CICP in the uphill-after-meal trial was higher than either of the two before-meal trials and the sedentary trial. The effect was similar for the downhill-after-meal trial except that it did not differ from the sedentary trial. In addition, the AUCs in the downhill-before-meal trial were different than its corresponding uphill trial (*t* = 3.83, *p* = 0.0028) and the sedentary trial (*t* = 5.15, *p* = 0.0003). Morning CICP AUCs also differed (*F*_(df 4,11)_ = 4.65, *p* = 0.0193, Figure 3 center and Table 3), with increases again attributable to the two exercise-after-meal trials compared to sedentary or before-meal trials. There were no significant changes in the afternoon CICP AUCs (Figure 3, right).

#### 3.3.2. Comparisons Between Exercise-After-Meal and Exercise-Before Meal Bone Marker AUCs (Table 3) 

**Table 3:** Statistical differences for CICP and CICP/CTX AUC group comparisons.

Variable	UAM vs. UBM	UAM vs. DBM	UAM vs. SED	DAM vs. UBM	DAM vs. DBM	DAM vs. SED
CICP AUCs, combined	*t* = 3.62, *p* = 0.0040	*t* = 7.45, *p* < 0.0001	*t* = 2.30, *p* = 0.042	*t* = 2.31, *p* = 0.0412	*t* = 6.14, *p* < 0.0001	NS
CICP AUCs AM	NS	*t* = 3.47, *p* = 0.0052	*t* = 2.98, *p* = 0.0125	NS	*t* = 2.85, *p* = 0.0158	*t* = 2.36, *p* = 0.038
CICP/CTX AUCs combined	*t* = 5.45, *p* = 0.0002	*t* = 7.57, *p* < 0.0001	*t* = 5.63, *p* = 0.0002	*t* = 6.03, *p* < 0.0001	*t* = 8.15, < 0.0001	*t* = 6.21, < 0.0001.
CICP/CTX AUCs AM	NS	*t* = 2.73, *p* = 0.0197	NS	*t* = 5.09, *p* = 0.0003	*t* = 5.99, *p* < 0.0001	*t* = 4.58, *p* = 0.0008
CICP/CTX AUCs PM	*t* = 5.96, *p* < 0.0001	*t* = 8.23, *p* < 0.0001	*t* = 7.26, *p* < 0.0001	NS	*t* = 3.62, *p* = 0.004	*t* = 2.84, *p* = 0.016

DF = 11 in all comparisons. UAM = uphill after meal trial; UBM = uphill before meal trial; DAM = downhill after meal trial; DBM = downhill before rmeal trial; SED = sedentary trial.

#### 3.3.3. CTX

**Figure 4:**
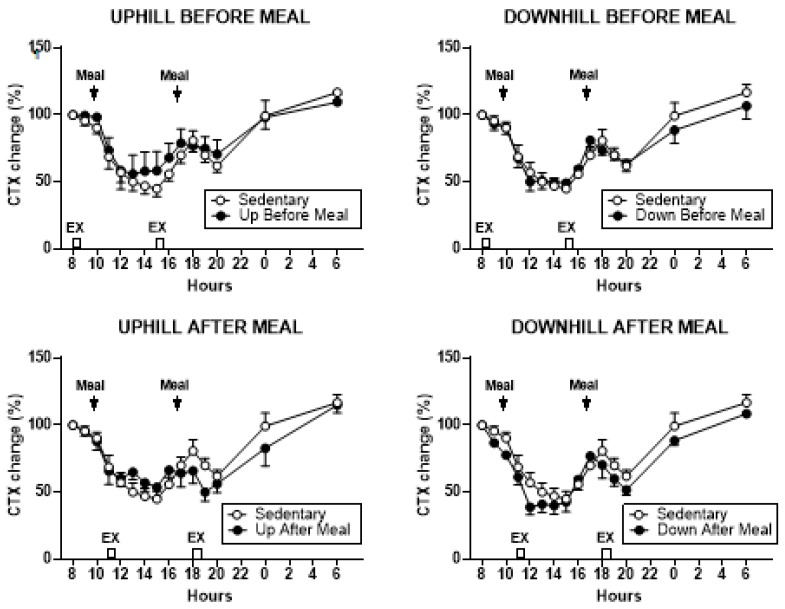
Percent changes in serum C-terminal telopeptide of type-I collagen (CTX) between sedentary trial and exercise trials performed before meals is shown at the top (left: UBM, right: DBM), and after exercise trials performed after eating, is shown at the bottom (left: UAM, right: DAM).

Treatment effects on percent CTX change are shown in Figure 4. CTX AUCs did not differ when the morning and afternoon AUCs were combined or examined individually.

#### 3.3.4. Osteogenic CICP/CTX Ratio

**Figure 5:**
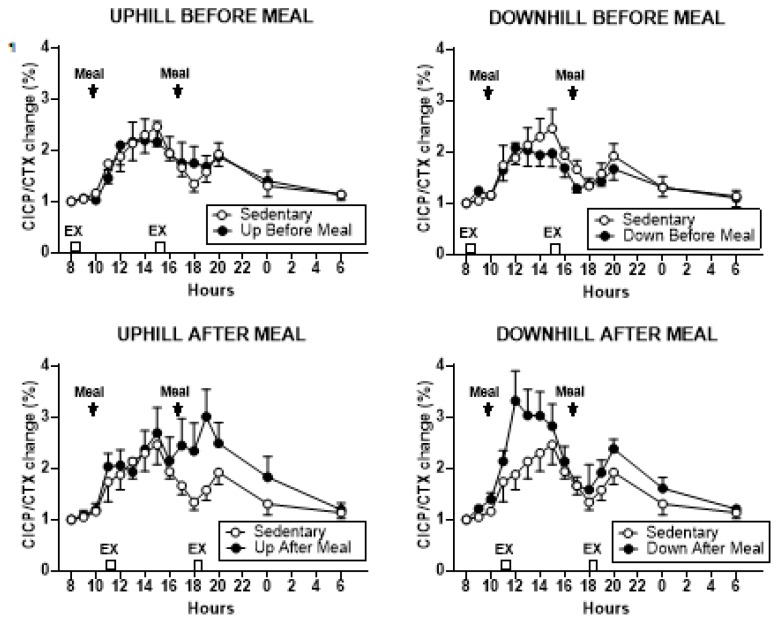
Percent changes in serum CICP/CTX ratio between sedentary trial and exercise trials performed before meals is shown at the top (left: UBM, right: DBM), and after exercise trials performed after eating, is shown at the bottom (left: UAM, right: DAM). CICP/CTX AUCs were significantly higher after second exercise bout after the meals in the UAM trial (bottom, left) and after first such bout in the DAM trial (bottom, right).

**Figure 6:**
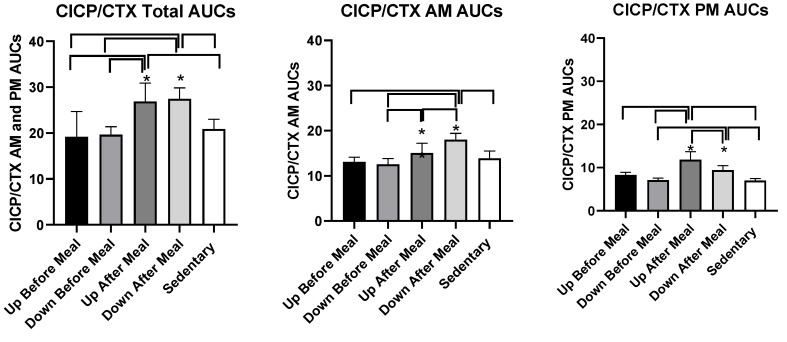
Postprandial CICP/CTX AUCs after the morning and afternoon meals combined (left) and after the morning (center) and afternoon (right) meals individually. Greatest increases in CICP/CTX AUCs were seen for the two exercise-after-meals trials (UAM and DAM) relative to sedentary trials both in the combined AUCs (left) and afternoon AUCs (right). The morning CICP/CTX AUCs remained higher only in the DAM relative to SED trial and two exercise-before-meals trials. Afternoon AUCs in UBM trial were also higher than in the DBM trial. * indicates significant difference relative to groups marked by the overhead bracket.

Treatment effects on the percent change in CICP/CTX ratio are shown in Figure 5, and the analysis of group differences in CICP/CTX AUCs in Figure 6 and Table 3. CICP/CTX ratio differed when the combined morning and afternoon AUCs were evaluated (*F*_(df 4,11)_ = 26.86, *p* < 0.0001). 

Group differences in AUCs followed the pattern seen in CICP AUCs with the two exercise-after-meal trials being higher than the two before-meal trials or the sedentary condition (Figure 6, left, Table 3). CICP/CTX ratio also differed when the morning AUCs (*F*_(df 4,11)_ = 10.90, *p* = 0.0008) and afternoon AUCs (*F*_(df 4,11)_ = 20.42, *p* < 0.0001) were considered separately. In the morning, the AUCs in the downhill-after-meal trial were higher than in both before-meal trials, the sedentary trial (Table 3, Figure 6, center, Table 3), as well as the uphill-after-meal trial (*t* = 3.26, *p* = 0.0076). The UAM trial had AUCs higher than the DBM trial (*t* = 2.73, *p* = 0.0197). The afternoon CICP/CTX ratios followed a similar pattern with the AUCs in two after-meal trials being higher than in both before-meal trials and the sedentary condition (Figure 6, right, Table 3). In addition, the afternoon AUCs in the downhill after-meal trial were higher than in the corresponding uphill trial (*t* = 4.65, *p* = 0.0007).

### 3.4. Glucose, Insulin, and HOMA-IR

#### 3.4.1. Glucose

**Figure 7:**
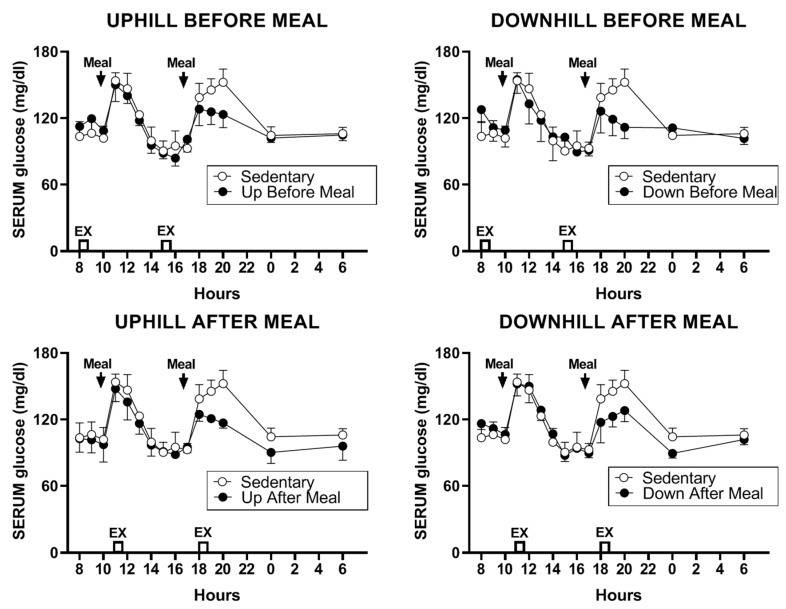
The change in serum glucose concentration in the four exercise relative to sedentary trials.

The change in serum glucose concentration in the four exercises relative to sedentary trials was marginally significant (F _(df 4,11)_ = 3.38, *p* = 0.049, Figure 7). Although plasma glucose was similarly lower during second postprandial period in all four exercise trials, this change attained significance only in three comparisons, between the downhill-before-meal trial and the two after-meal trials (UAM: *t* = 3.40, *p* = 0.0059; DAM: *t* = 2.71, *p* = 0.0204) and the uphill-before-meal trial (*t* = 2.58, *p* = 0.0258).

#### 3.4.2. Insulin

**Figure 8:**
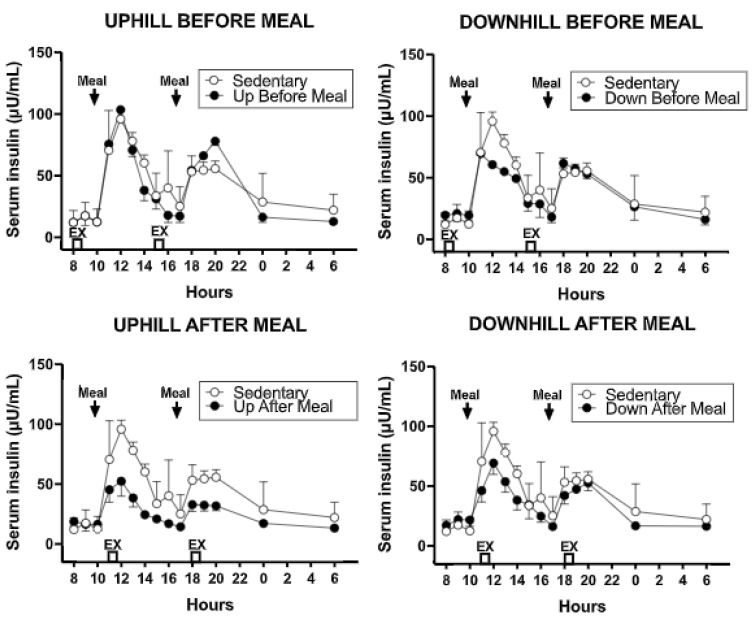
The changes in serum insulin concentration in the four exercise relative to sedentary trials. The largest insulin decline occurred in the UAM trial relative to sedentary and exercise-before-meal trials.

**Figure 9:**
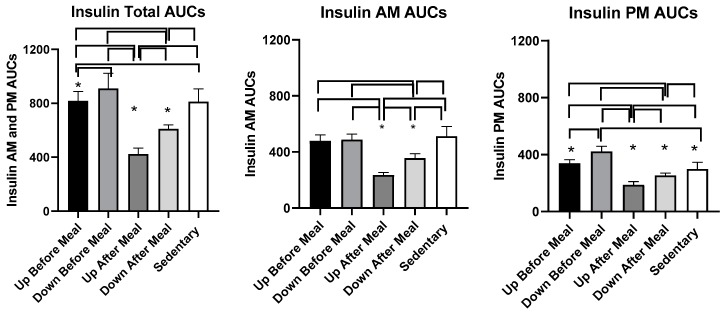
Postprandial insulin AUCs after the morning and afternoon meals combined (left) and after the morning (center) and afternoon (right) meals individually. Greatest decreases in insulin AUCs were in the exercise-after-meal trials (UAM) relative to sedentary trials and relative to exercise-before-meals trials in combined (left) as well as individual AUCs (center and right). In addition, within the exercise-after-meal condition, afternoon UAM AUC was lower than the DAM AUC. * indicates significant difference relative to groups marked by the overhead bracket.

The overall effect of exercise was to reduce PP serum insulin concentration relative to the sedentary trial (Figure 8). This effect was also apparent in combined AUCs (*F*_(df 4,11)_ = 69.96, *p* < 0.0001, Figure 9 and Table 4) and also during morning (*F*_(df 4,11)_ = 41.11, *p* = < 0.0001) and afternoon PPs (*F*_(df 4,11)_ = 47.01, *p* = < 0.0001). 

#### 3.4.3. Comparisons Between Exercise-After-Meal and Exercise-Before Meal Insulin and HOMA-IR AUCs (Table 4) 

Combined insulin AUCs were lower in both exercise-after-meal trials than in the two before-meal trials or than in sedentary condition (Figure 9, left, Table 4). In addition, insulin AUCs in the uphill-after-meal trial was lower than in the corresponding downhill trial (*t* = 5.61, *p* = 0.0002). Uphill before-meal trial had lower insulin AUCs than the downhill-before meal trial (*t* = 2.82, *p* = 0.0166) and sedentary trial (*t* = 3.0, *p* = 0.0122). A similar pattern of differences as in combined insulin AUCs carried over to the morning AUCs. The AUCs in after-meal trials were lower than the AUCs in the corresponding before-meal or sedentary trials (Figure 9, center, Table 4). The UAM morning insulin AUC was also lower than in DAM trial (*t* = 4.67, *p* = 0.0007). An almost identical pattern of insulin AUC change to that seen with combined AUCs was apparent in the afternoon (Figure 9, right, Table 4) with the AUCs in two after-meal trials lower than in the before-meal and sedentary trials. In addition, UAM afternoon insulin AUC was lower than in DAM trial (*t* = 3.57, *p* = 0.0044) and the DBM AUCs were lower than in UBM (*t* = 4.62, 0.0007) and SED (*t* = 6.79, *p* < 0.0001) trials.

#### 3.4.4. HOMA-IR Measure of Insulin Resistance

**Figure 10:**
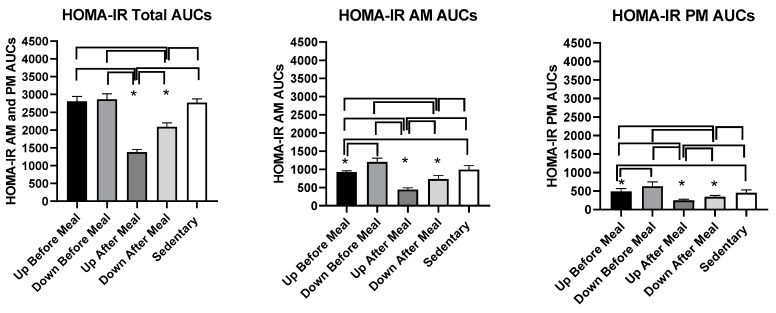
Postprandial homeostatic model (HOMA-IR) AUCs after the morning and afternoon meals combined (left) and after the morning (center) and afternoon (right) meals individually. A consistent reduction in HOMA-IR AUCs was seen only for the UAM exercise-after-meal trial in comparison to SED, two exercise-before-meal trials, and in the afternoon also relative to DAM, the other exercise-after-meal trial. * indicates significant difference relative to groups marked by the overhead bracket.

Changes in glucose and insulin concentrations served to calculate changes in insulin resistance using the HOMA-IR procedure (Figure 10). HOMA-IR AUC results were influenced by the pattern of insulin results. There was an overall treatment effect for combined (*F*_(df 4,11)_ = 177.33, p ≤ 0.0001), morning (*F*_(df_ =_4,11)_ =65.92, *p* ≤ 0.0001), and afternoon (*F*_(df 4,25)_ = 54.24, *p* ≤ 0.0001) HOMA-IR AUCs (Figure 10, and Table 4). Combined (Figure 10, left and Table 4), as well as individual morning (Figure 10, center) and afternoon AUCs (Figure 10, right), were lower in both after-meal trials relative to before-meal and sedentary trials. Also, in all three comparisons, HOMA-IR AUC in the uphill after-meal trial was lower than in the corresponding downhill trial (*t* = 10.7, *p* < 0.001 in combined AUCs; *t* = 5.82, *p* = 0.0001 in AM AUCs, and *t* = 3.02, *p* = 0.0116). The morning and afternoon HOMA-IR AUCs deviated from the combined analysis in that in both cases, UBM trial had lower AUC than the DBM and sedentary trials (AM: *t* = 5.68, *p* = 0.0001 vs. DBM, *t* = 4.19, *p* = 0.0015 vs. SED; PM: *t* = 4.99, *p* = 0.0004 vs. DBM, *t* = 7.54, *p* ≤ 0.0001 vs. SED).

### 3.5. Hormone Measurements

#### 3.5.1. PTH

**Figure 11:**
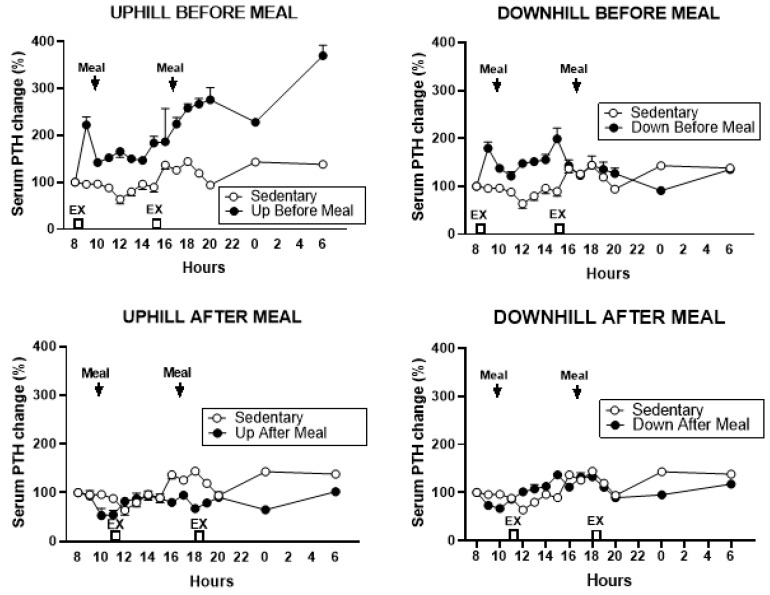
Percent changes in serum parathyroid hormone (PTH) between sedentary trial and exercise trials performed before meals is shown at the top (left: UBM, right: DBM), and after exercise trials performed after eating, is shown at the bottom (left: UAM, right: DAM). PTH response was higher after both exercise trials performed before eating than during the sedentary trial and did not change during two exercise-after-meal trials.

There was a difference in the total PTH AUCs in the five trials (*F*_(df = 4,4)_ = 26.68, *p* = 0.0038). PTH increased in two exercise-before-meal trials relative to the two exercise-after-meal trials and sedentary trial (Figure 11).

**Figure 12:**
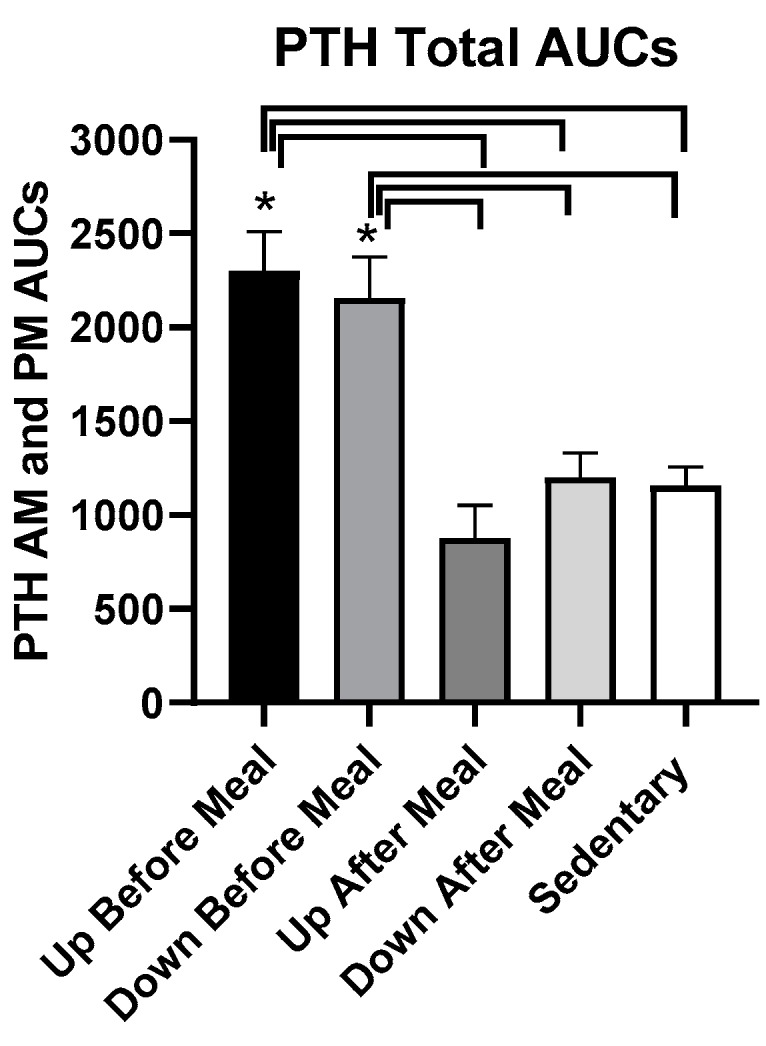
Total postprandial PTH AUCs in the five trials. AUCs in the two exercise-before-meal trials were significantly higher than in the SED trial, and total PTH AUC in the DAM exercise-after-meal trial was significantly higher than in the UAM trial. PTH AUCs in the morning and afternoon postprandial periods were not significantly different. * indicates significant difference relative to groups marked by the overhead bracket.

This pattern of change was confirmed in the total postprandial AUC analysis (Figure 12). PTH AUCs were higher in both before-meal trials relative to two after-meal and sedentary trials (UBM vs. UAM: *t* = 7.95, *p* = 0.0014, vs. DAM: *t* = 6.61, *p* = 0.0027, vs. SED: *t* = 6.41, *p* = 0.003; DBM vs. UAM: (*t* = 7.15, *p* = 0.002, vs. DAM: *t* = 5.77, *p* = 0.0045, and vs. SED: *t* = 5.61, *p* = 0.005). 

#### 3.5.2. Cortisol

**Figure 13:**
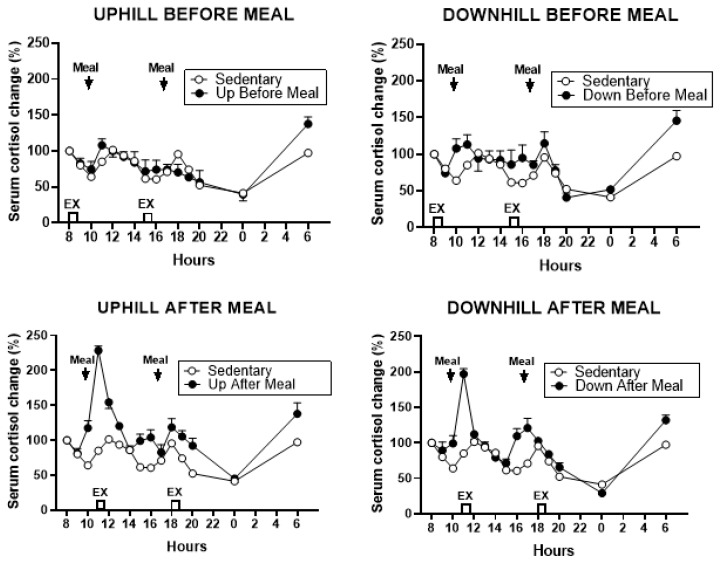
Percent changes in serum cortisol between sedentary trial and exercise-before-meal trials is shown at the top (left: UBM, right: DBM), and after exercise trials performed after eating, is shown at the bottom (left: UAM, right: DAM).

Cortisol response increased during the two exercise-after-meal trials, more after the first than after the second, postprandial period (Figure 13.)

**Figure 14:**
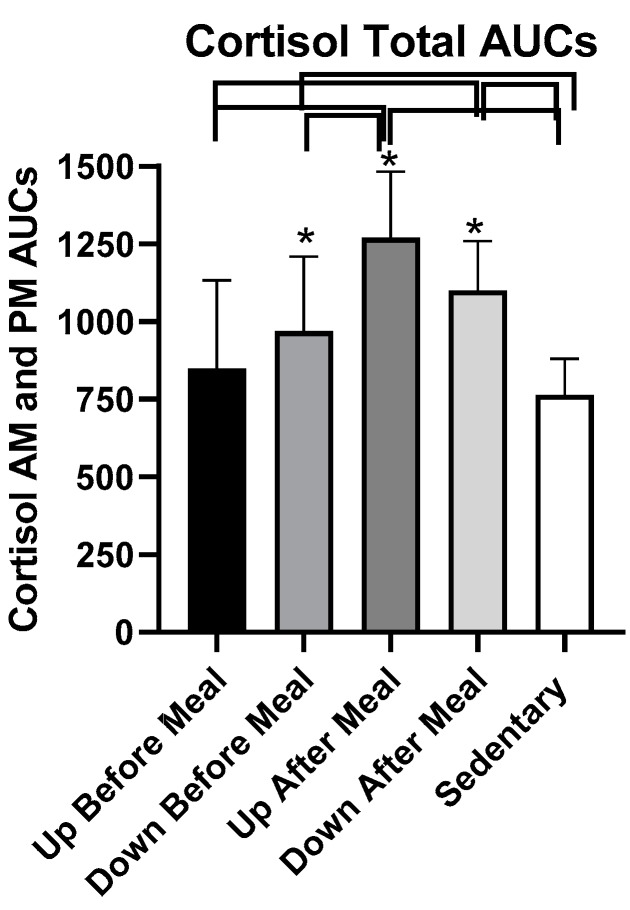
Total postprandial cortisol AUCs in the five trials. The AUCs in the two exercise-after-meals trials were higher than in the SED trial, and combined cortisol AUC in the UAM exercise-after-meal trial was higher than the exercise-before-meal UBM trial. There were no treatment differences within morning and afternoon cortisol AUCs. * indicates significant difference relative to groups marked by the overhead bracket.

Total cortisol AUCs differed for the five trials (*F*_(df 4,25)_ = 10.62_,_ *p* < 0.0001, Figure 14) largely due to higher cortisol responses in the two after-meal trials compared to the two before-meal and sedentary trials. UAM AUCs were higher than UBM (*t* = 4.77, *p* = 0.0006), DBM (*t* = 3.51, *p* = 0.0048), and SED (*t* = 5.78, *p* = 0.0001) AUCs. A similar pattern was obtained in DAM trial where the AUCs were higher than in UBM (*t* = 2.9, *p* = 0.0144) and in SED (*t* = 3.92, *p* = 0.0024) trials. Total cortisol AUC was also higher in DBM trial compared to the SED trial (*t* = 2.27, *p* = 0.0444).

## 4. Discussion

We designed this study with the core assumption, expressed in our first hypothesis, that the increased mechanical loading of downhill exercise would significantly increase the osteogenic CICP/CTX ratio. This expectation was based on a previous study where healthy postmenopausal women walked 4.8 km per day 4 days a week for 15 weeks, and preserved, or increased, the areal BMD of their legs and whole body, but not of other skeletal sites. This effect required walking at higher relative intensity of 75% of maximal effort, while the same volume of walking at the relative intensity of 46% did not prevent BMD losses. GRFs measured in the laboratory on a force plate for three walking speeds revealed that mechanical loading had to exceed 872 N (1.22 times body weight) to prevent BMD losses or produce BMD increases [24]. While this previous study was done with healthy postmenopausal women, and walking was on a level surface, the absolute GRFs generated with downhill walking in the present study were well in excess of that putative threshold (1104.72 ± 005 N in DBM trial, and 1105.0 ± 0.07 N in DAM trial, Table 2), and the same was true for GRFs normalized by weight (1.59 ± 0.05 N/kg in DBM trial and 1.64 N/kg in DAM trial, Table 2). While walking uphill generated absolute and relative GRFs at about this putative osteogenic loading threshold (857.50 ± 52.8 N and 1.31 ± 0.02 N/kg in UBM and 795 ± 36.5 N and 1.12 ± 0.04 N/kg, respectively in UAM trial, Table 2), we expected at least an intensity dose–response effect on the bone marker response in the present study. Instead, the intensity of mechanical loading had no effect on the observed changes in CICP, the marker of bone formation (Figure 2 and Figure 3) and in the osteogenic CICP/CTX, the ratio between the markers of formation and resorption (Figure 5 and Figure 6). Therefore, our first hypothesis was not supported.

Against our expectation, expressed in hypothesis 2, that the loading stimulus of exercise would produce an osteogenic effect that would extend for at least 80 pre-meal minutes through the immediate postprandial period, as many exercise effects are regularly seen when performed in either fasted or postprandial state, increases in CICP (Figure 2 and Figure 3) and the osteogenic CICP/CTX ratio (Figure 5 and Figure 6) were manifested in this study only when exercise occurred in the PP. This finding refuted our second hypothesis. It would appear that bone anabolic response is not only very briefly sensitive to purely mechanical stimulation [25,26] followed by hours-long refractory period to such stimulation [25,27,28], but that the osteogenic response appears to be restricted and enhanced by nutrient intake during a brief postprandial period. In our study, this nutrient-and-exercise period of osteogenic sensitivity occurred only during the first postprandial period of downhill exercise, DAM (Figure 5, bottom, right) and only during the second postprandial period of uphill exercise, UAM (Figure 2 and Figure 5, bottom left). 

We speculate that the appearance of the increase in CICP and the CICP/CTX ratio after the first episode of meal eating followed by downhill exercise, but not after the second such episode, is a consequence of the operation of the already described refractory period to repeat mechanical stimulation [25,27,28]. The delayed appearance of CICP/CTX rise to uphill exercise following the second meal may be related to greater energy expenditure and muscle glucose uptake during uphill, compared to lower-intensity downhill, exercise. If that was the case, we speculate that muscle tissue may have initially outcompeted the bone tissue for glucose uptake.

Our third hypothesis was that the osteogenic response would predominantly reflect a decline in the marker of bone resorption rather than an increase in the marker of bone formation. This hypothesis was based on the evidence that meal eating lowers circulating concentrations of CTX compared to its concentrations during overnight or diurnal fast [22]. Additional support for the third hypothesis is the findings that the meal-associated rise in the gut hormone GLP-2 occurs concomitantly with a decline in CTX concentration, and there is evidence that GLP2 administration reduces the circulating level of this resorption marker [22]. We were, therefore, surprised to see no effect of exercise and meal timing on CTX concentration in this study (Figure 4) and hence, our third hypothesis is also refuted.

The unexpected evidence that the skeleton of diabetic postmenopausal women is capable of osteogenic response is surprising in view of the general expectation of reduced osteogenic response in the aging skeleton [10,11]. Additional evidence for reduced bone quality in T2D [9] made the expectation that our experimental paradigm could increase osteogenesis in postmenopausal diabetic women even less probable. Examination of changes in metabolites, insulin, cortisol, and PTH provide grounds for an attempt at interpreting the probable cause of the observed osteogenic response. A hypothesis that we did not explicitly make, but which guided our hormone and metabolite measurements and calculations of the HOMA-IR assessment of insulin resistance, was that depressed osteogenesis in diabetes may reflect reduced access of bone tissue to nutrient energy necessary to respond to a mechanical stimulus. While we observed no trial-specific change in plasma glucose (Figure 7), we found significant declines in postprandial insulin during two exercise-after-meal trials but not after either of the two exercise-before meal trials. HOMA-IR AUC was particularly reduced in the uphill post-meal exercise UAM relative to sedentary trial, but not in the two exercise-before-meal trials. Reduced HOMA-IR AUC measure of insulin resistance in the two exercise-after-meal trials, with no change in corresponding glucose concentration, allows the inference that when exercise took place shortly after eating, there was an increase in glucose uptake by muscle, and possibly also by bone. 

Changes in circulating cortisol, which also increased during the exercise-after-meal trials (Figure 13 and Figure 14), support the inferred hypothesis that our observation of increased CICP and osteogenic CICP/CTX ratio in exercise-after-meal trials may reflect increased access of the diabetic postmenopausal bone tissue to nutrients. Uptake of glucose in particular may have played a role because the diet we provided contained 50% carbohydrate. In addition, the well-known increase in plasma cortisol during the mid-day meal [39,40] is facilitated by high-glucose diet [40]. Most of the increase in meal-associated cortisol is of adrenal origin, but the hormone facilitates additional hepatic production of cortisol from cortisone by activating type 1 11ß-hydroxysteroid dehydrogenase [40]. One of the functions of cortisol in stress (and upon rising in the morning) is to increase hepatic glucose production by gluconeogenesis and to allow its release into circulation [41]. While long-term systemic exposure to cortisol is known to cause bone resorption [34], the concurrent increases in osteogenic index and cortisol in exercise-after-meal trials in this study indicate that short-term increases in serum cortisol during post-meal exercise serve functions other than bone resorption. Cortisol secretion is also amplified by exercise in conjunction with its meal-triggered release [42]. It is therefore plausible that increases in circulating cortisol observed in the UAM trial in our study were facilitated by reduced insulin resistance and high dietary carbohydrate content in combination with post-meal exercise, and that this increase in cortisol augmented glucose supply to muscle and the bone tissue. 

We also observed an increase in circulating PTH after exercise that preceded the meals. The increase was closer to a pulse in the DBM trial as it was confined only to the morning PP period. In UBM trial, PTH rise was extended for 22 h after the first exercise bout. It is therefore difficult to speculate whether either pattern of release had any impact on osteogenic response, a positive effect expected with the pulsatile pattern [28], or interference with this response with protracted PTH release seen as secondary hyperparathyroidism of postmenopausal women [43].

This study has some limitations. We were able to recruit only 15 diabetic postmenopausal women and had to assign them to two trials, each, to achieve an acceptable statistical power. A larger number of subjects would provide additional confidence in the repeatability of our results. Hemoglobin A1c was not measured to provide an assessment of diabetic control. Instead, the medical history, anti-diabetic and glucose-lowering medication use, and high fasting plasma glucose values documented the subjects’ diabetic status. The statistical power is particularly the case with PTH measurements where only four subjects per group were used. We also did not collect blood samples during the afternoon PP period as frequently as during the morning one, making the comparisons between the two PPs uneven. We confounded cardiorespiratory and mechanical stress, as both variables were changed concurrently rather than independently. However, the psychosomatic stress of exercise in this study was not significantly affected as shown by a lack of group differences in either the RPE or heart-rate scores (Table 2). Along with simultaneous manipulation of cardiorespiratory and mechanical stress, the energy expenditure in uphill and downhill trials were not of equal magnitude. Despite these limitations, the interpretation in favor of our inference that exercise after eating is osteogenic in diabetic postmenopausal women appears convincing on the strength of our data.

## 5. Conclusions

In conclusion, our novel finding is that providing an effective exercise stimulus shortly after eating promotes osteogenesis in diabetic postmenopausal women. The effect was entirely the result of post-meal timing of exercise and unaffected by significant differences in applied mechanical loads. The demonstration that the skeleton in postmenopausal diabetic women is capable of responding to exercise and nutrients with increases in a marker of bone formation opens the possibility of using this lifestyle paradigm as a substitute or a complement to the commonly used anti-resorptive medication. It even provides encouragement for development of osteogenic, as opposed to predominantly anti-resorptive, pharmacological approaches. 

## Figures and Tables

**Table 4:** Statistical differences for insulin and HOMA-IR AUC group differences.

Variable	UAM vs UBM	UAM vs DBM	UAM vs SED	DAM vs UBM	DAM vs DBM	DAM vs SED
Insulin AUCs, combined	*t* = 11.86, *p* ≤ 0.0001	*t* = 14.68, *p* ≤ 0.0001	*t* = 11.69, *p* ≤ 0.0001	*t* = 6.25, *p* ≤ 0.0001	*t* = 9.07, *p* ≤ 0.0001	*t* = 6.08, *p* ≤ 0.0001
Insulin AUCs, AM	*t* = 9.43, *p* ≤ 0.0001	*t* = 9.80, *p* ≤ 0.0001	*t* = 10.75, *p* ≤ 0.0001	*t* = 4.76, *p*=0.0006	*t* = 5.13, *p* = 0.0003	*t* = 6.09, *p* ≤ 0.0001
Insulin AUCs, PM	*t* = 8.25, *p* ≤ 0.0001	*t* = 12.87, *p* ≤ 0.0001	*t* = 6.08, *p* ≤ 0.0001	*t* = 4.68, *p*=0.0007	*t* = 9.30, *p* ≤ 0.0001	*t* = 2.51, *p* = 0.029
HOMA-IR AUCs, combined	*t* = 20.91, *p* ≤ 0.0001	*t* = 22.12, *p* ≤ 0.0001	*t* = 20.52, *p* ≤ 0.0001	*t* = 10.49, *p* ≤ 0.0001	*t* = 11.42, *p* ≤ 0.0001	*t* = 9.97, *p* ≤ 0.0001
HOMA-IR AUCs, AM	*t* = 9.54, *p* ≤ 0.0001	*t* = 15.22, *p* ≤ 0.0001	*t* = 11.02, *p* ≤ 0.0001	*t* = 3.72, *p* = 0.0034	*t* = 9.40, *p* ≤ 0.0001	*t* = 5.20, *p* = 0.0003
HOMA-IR AUCs, PM	*t* = 8.54, *p* ≤ 0.0001	*t* = 13.78, *p* ≤ 0.0001	*t* = 6.04, *p* ≤ 0.0001	*t* = 5.73, *p* = 0.0001	*t* = 10.66, *p* ≤ 0.0001	*t* = 3.12, *p* = 0.0098

DF = 11 in all comparisons.; UAM = uphill after meal trial; UBM = uphill before meal trial; DAM = downhill after meal trial; DBM = downhill before rmeal trial; SED = sedentary trial.
